# Supplementary material for: Visual Detection of Clostridium perfringens Alpha Toxin by Combining Nanometer Microspheres with Smart Phones
Source: Microorganisms. 2020 Nov 26;8(12):1865. doi: 10.3390/microorganisms8121865 (PMC7761010; doi:10.3390/microorganisms8121865)
Supplement: Supplementary file 1 [file microorganisms-08-01865-s001.pdf]

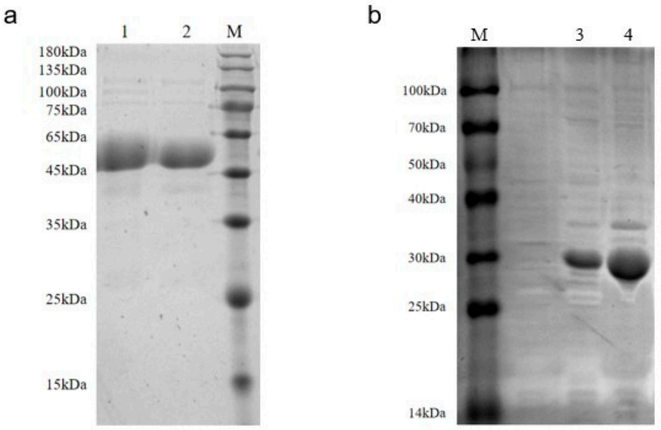


**Figure S1.** SDS-PAGE results of expression and purification of CPA recombinant proteins $CPA_{C3}$ and CPA. (**a**) M: protein marker (GenStar M221), lane 1–2: purified recombinant $CPA_{C3}$ protein; (**b**) M: protein marker (Blue Plus) lane 3–4: purified recombinant $CPA_{N}$ protein.

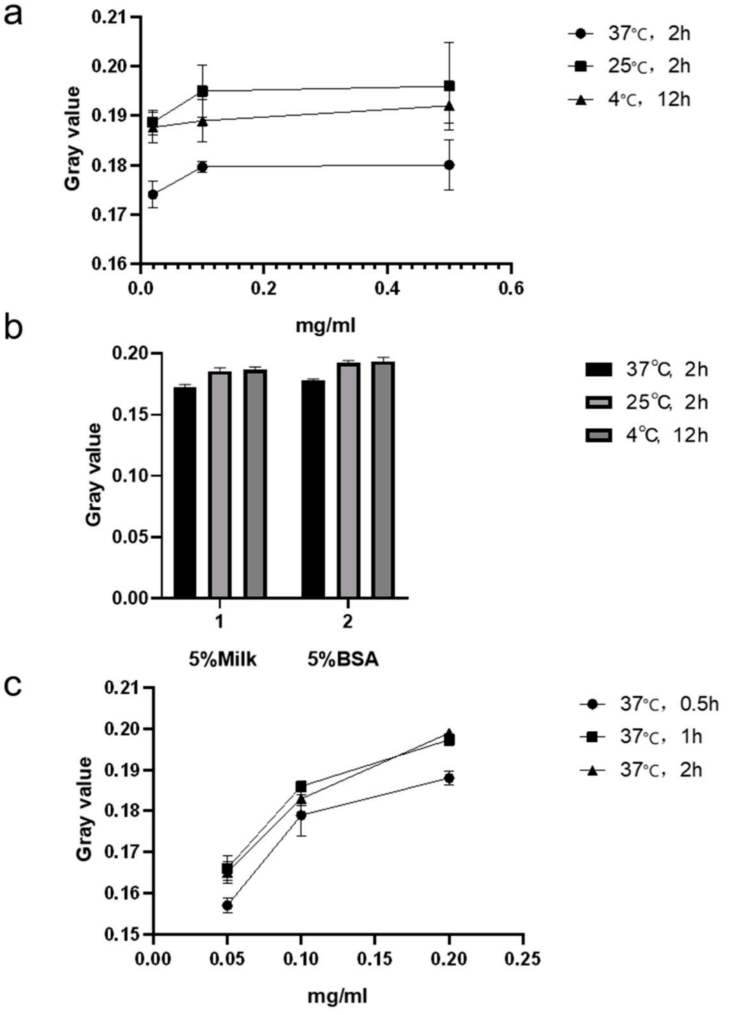

**Figure S2.** Optimal conditions for microsphere fluorescence detection experiment. (**a**) The optimal coating concentration of CPA$_{C3}$ antibody to microspheres under the fixed capture concentration of 10 LD$_{50}$ CPA were determined by variable incubation time, temperature, and then photograph fluorescence imaging through observation of fluorescence microscope, and continue to get the specific value through the gray value analysis. (**b**–**c**) Similarly, the optimal blocking conditions of coating of fluorescence microscope, the dilution concentration (1:50, 1:100, 1:200 dilution of FITC-anti-CPA$_{N}$ antibody conjugate), incubation time of FITC-labeled CPA$_{N}$ antibody conjugate were also determined under a different blocking solution (5% skim milk-PBST-20, 5% BSA PBST-20 buffer) conditions. Triplicates were performed under different conditions.

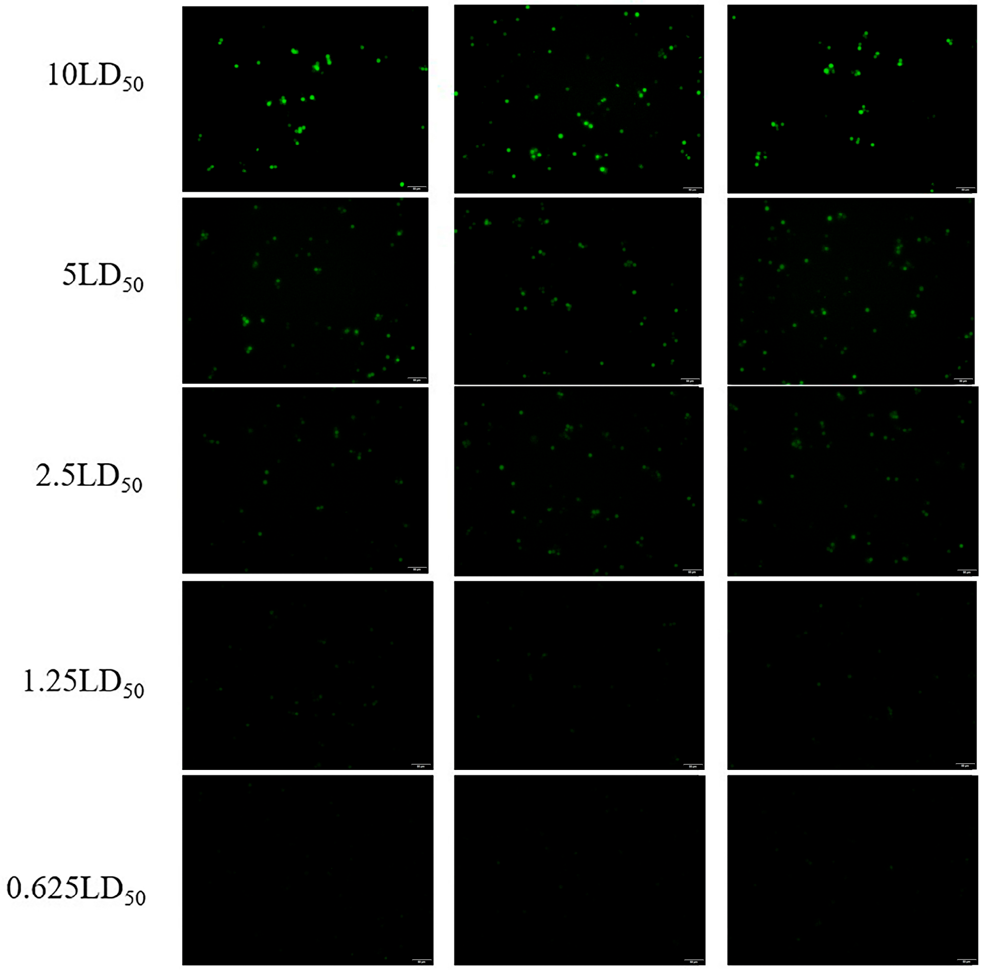


**Figure S3.** Fluorescence pictures under different toxin concentrations. From top to bottom, the concentrations are 10LD$_{50}$, 5LD$_{50}$, 2.5LD$_{50}$, 1.25LD$_{50}$, respectively, 0.625LD$_{50}$. Three biological replicates for each concentration. Scale bar = 50 μm.

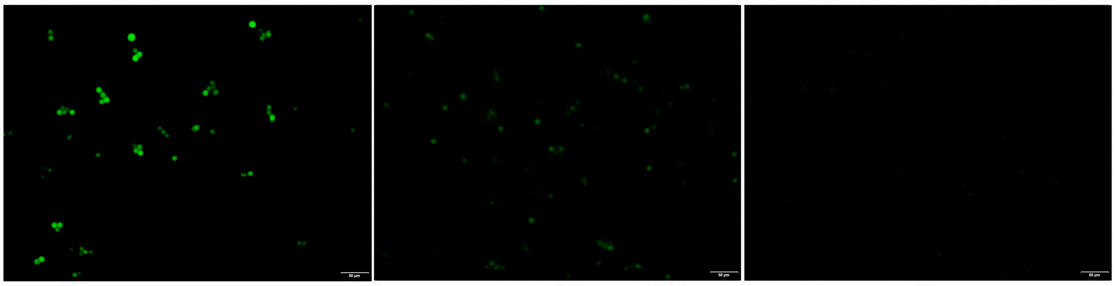

**Figure S4.** Fluorescence pictures of different toxin concentrations in milk samples. The concentrations from left to right are $10LD_{50}$, $2.5LD_{50}$, and $0.625LD_{50}$, respectively. Scale bar = 50 μm.

**Table S1.** Determination of optimal antibody coating conditions (fix other conditions unchanged; choose toxin concentration of 10LD50, 3 biological replicates).

| | Average Gray Value | | |
|---|---|---|---|
| Temperature time | 0.02mg/mL Capture antibody | 0.1mg/mL Capture antibody | 0.1mg/mL Capture antibody |
| 37 °C, 2h | 0.174 | 0.1795 | 0.18 |
| 25 °C,2h | 0.1885 | 0.195 | 0.196 |
| 4 °C12h | 0.1875 | 0.189 | 0.192 |

**Table S2.** Determination of optimal blocking conditions (fixing other conditions unchanged, choosing a toxin concentration of 10LD50, 3 biological replicates).

| | Average Gray Value | |
|---|---|---|
| Temperature time | 5%Skimmed milk powder | 5%BSA |
| 37 °C, 2 h | 0.172 | 0.178 |
| 25 °C, 12 h | 0.1855 | 0.192 |
| 4 °C, 12 h | 0.187 | 0.1935 |

**Table S3.** The optimal incubation time and dilution of the fluorescence-labeled detection antibody (the concentration of detection antibody is 10 mg/mL and make 50, 100, 200-fold dilution; fixing other conditions unchanged, selecting the toxin concentration of 10LD50, 3 biological replicates).

| | Average Gray Value | | |
|---|---|---|---|
| Temperature time | 1:50 | 1:100 | 1:200 |
| 37 °C, 30 min | 0.188 | 0.179 | 0.157 |
| 37 °C, 1 h | 0.197 | 0.186 | 0.166 |
| 37 °C, 2 h | 0.199 | 0.183 | 0.165 |

**Table S4.** The gray value of the fluorescence images of different concentrations of alpha toxin.

| LD50 | Gray Value (Triplicate) | | | Mean | SD |
|---|---|---|---|---|---|
| 10 | 0.202 | 0.193 | 0.191 | 0.202 | 0.193 |
| 5 | 0.097 | 0.098 | 0.091 | 0.097 | 0.098 |
| 2.5 | 0.054 | 0.053 | 0.053 | 0.054 | 0.053 |
| 1.25 | 0.029 | 0.024 | 0.028 | 0.029 | 0.024 |
| 0.625 | 0.016 | 0.012 | 0.015 | 0.016 | 0.012 |
